# Gonadotropin-Releasing Hormone (GnRH) and Its Agonists in Bovine Reproduction II: Diverse Applications during Insemination, Post-Insemination, Pregnancy, and Postpartum Periods

**DOI:** 10.3390/ani14111575

**Published:** 2024-05-26

**Authors:** Eman M. Hassanein, Zoltán Szelényi, Ottó Szenci

**Affiliations:** 1 Department of Obstetrics and Food Animal Medicine Clinic, University of Veterinary Medicine Budapest, H-2225 Üllő, Hungary; em.mostafa@alexu.edu.eg (E.M.H.); szelenyi.zoltan@univet.hu (Z.S.); 2 Animal and Fish Production Department, Faculty of Agriculture, Alexandria University, Alexandria 21545, Egypt

**Keywords:** GnRH analog, repeat breeder, embryo mortality, cystic ovarian disease, embryo transfer, nano-drug delivery system, reproductive performance, dairy cattle

## Abstract

**Simple Summary:**

Simple Summary: This review underscores the positive impact of Gonadotropin-releasing hormone (GnRH) and its agonists across several physiological stages, including estrous synchronization, post-insemination, pregnancy, and the postpartum period. It focuses on their role in overcoming reproductive dysfunctions such as repeat breeder cows, early embryonic loss, and cystic ovarian syndrome. The review also highlights their influence on enhancing the productivity of embryo transfer programs. Additionally, it introduces the emerging field of nano-drug delivery systems for GnRH agonists, highlighting potential benefits. The review aims to improve reproductive efficiency and health management in dairy cattle by emphasizing the critical need for further research and development.

**Abstract:**

The administration of GnRH and its agonists benefits various aspects of bovine reproductive programs, encompassing physiological stages such as estrous synchronization, post-insemination, pregnancy, and the postpartum period. The positive impact of GnRH administration in overcoming challenges like repeat breeder cows, early embryonic loss prevention, and the management of cystic ovarian disease (COD) is thoroughly surveyed. Furthermore, this review focuses on the significance of GnRH administration during the postpartum period, its role in ovulation induction, and how it enhances the productivity of embryo transfer (ET) programs. An emerging feature of this field is introduced, focusing on nano-drug delivery systems for GnRH agonists, and the potential benefits that may arise from such advancements are highlighted. While this review offers valuable insights into various applications of GnRH in bovine reproduction, it emphasizes the crucial need for further research and development in this field to advance reproductive efficiency and health management in dairy cattle.

## 1. Introduction

**Figure 1:**
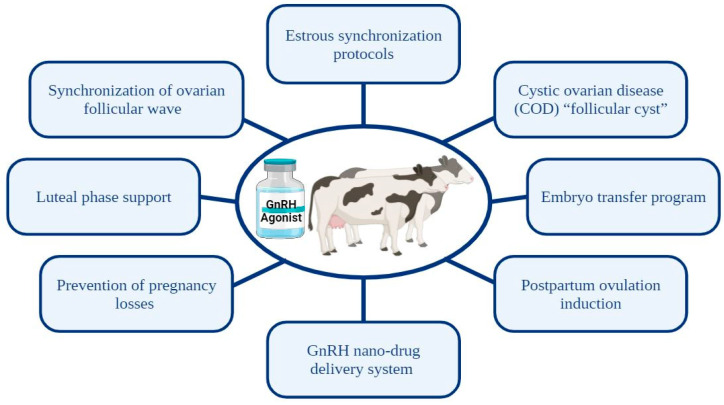
The diagram illustrates the various applications of Gonadotropin-releasing hormone (GnRH) and its agonists in bovine reproductive practices.

In bovine reproductive programs, GnRH and its agonists play a pivotal role in regulating various aspects of reproduction throughout estrous synchronization, post-insemination, pregnancy, and the postpartum periods (Figure 1). We have recently reviewed the chemical structure of GnRH agonists and their role in the biosynthesis of gonadotropins by the pituitary. This is important for regulating the reproductive process, specifically emphasizing their application in estrous synchronization [1]. This review article illustrates the diverse applications of GnRH agonists across different physiological stages.

Administering GnRH during artificial insemination (AI) is valuable for repeat breeder cows, as it significantly enhances pregnancy maintenance. This effect is more pronounced in repeat-breeding dairy cows than in their first postpartum insemination, leading to increased conception rates and improved fertility [2]. The administration of GnRH has a positive impact on preventing early embryonic mortality in cattle. Approximately 25% of bovine embryos face non-viability within the initial three weeks of gestation due to premature luteolysis and a decrease in progesterone (P_4_) concentration, which can be addressed by GnRH administration [3].

Injecting GnRH agonists during the beginning of the diestrous phase (days 4 to 9) after AI contributes to sustained luteinization of follicles, potentially boosting embryo survival percentages [4,5]. The role of GnRH in delaying luteolysis and influencing follicular dynamics contributes positively to increased conception rates [6,7]. However, the impact of GnRH agonists during the diestrus (days 11 to 14) after AI varies among studies, influenced by factors such as synchronization programs, heat stress, and environmental conditions [8,9,10]. On the other hand, it is noteworthy that administering GnRH around day 30 after AI didn’t have a significant effect on maintaining pregnancy [11].

In managing cystic ovarian disease (COD), specifically “follicular cyst”, GnRH administration emerges as a preferred treatment [4]. Studies highlight its efficacy in inducing follicular dynamic alterations and contributing to the remission of ovarian cysts [12]. Combining GnRH agonists with other hormones and modified breeding programs is recommended for improved therapeutic effects [13]. 

In the critical postpartum period (within 50 to 60 days postpartum) for dairy herds, hormonal treatments, including GnRH, are employed to synchronize reproductive functions and induce ovulation [14]. In beef herds, administering GnRH treatment during the postpartum period to induce ovulation of the first postpartum dominant follicle (DF) during the plateau/early declining phase of DF growth enhances ovarian functions. It reduces calving-to-estrous intervals, thereby positively influencing reproductive performance [15].

In ET programs involving both in vivo and in vitro-derived embryos, there is a focus on enhancing reproductive efficiency. GnRH, human chorionic gonadotropin (hCG), and their analogs have demonstrated positive outcomes, including increased pregnancy rates and reduced embryonic losses [16,17,18,19].

Furthermore, the emerging field of nano-drug delivery systems for GnRH agonists presents a promising avenue, with studies indicating potential benefits, including reduced doses, enhanced bioavailability, and improved fertility outcomes in different farm animals [20,21]. However, further research is needed to fully understand its effectiveness, safety, and economic feasibility in dairy cattle.

In summary, this review provides insights into the multifaceted applications of GnRH and its agonists in bovine reproductive practices, highlighting their diverse roles in optimizing conception rates, preventing pregnancy losses, and overcoming reproductive challenges across various stages.

## 2. Enhancing Ovulation Response and Conception Rates at the Onset of Estrus and AI Time

Although fixed-time artificial insemination (FTAI) is widely used in dairy reproductive management programs, estrous detection remains essential. Increased physical activity serves as a secondary indicator of estrus in dairy cattle. Recently, electronic systems such as automatic activity monitoring (AAM) [22] and accelerometer systems [23] have been developed to monitor physical activity continuously. This helps to predict the timing of AI in the dairy industry. Variations in the duration of estrus and the timing of AI relative to ovulation can lead to decreased fertility in some cows. However, AI timing based on activity monitoring systems was acceptable for most cows showing increased activity [24]. Studies have shown that administering GnRH at the onset of behavioral estrus (OE) positively affects pregnancy rates by increasing ovulation responses or reducing variation in the interval to ovulation [25]. Therefore, several studies recommended utilizing GnRH at the estrous phase to elevate P_4_ concentrations during the luteal phase of the subsequent estrous cycle. Table 1 presents findings from various studies that support this recommendation.

In a study conducted during the winter season, it was observed that the administration of GnRH and agonists resulted in enhanced pregnancy rates when given at the time of OE, while no beneficial effects were noted when GnRH was administered later in the period of behavioral estrus [26]. Moreover, in cows administered GnRH at the OE, the amplitude of the spontaneous luteinizing hormone (LH) pre-ovulatory release was twice as great as typically detected compared to a second, lesser amplitude induction of LH release when administered later in the period of behavioral estrus. Another study conducted during the summer months demonstrated increased pregnancy rates in dairy cows administered GnRH when detecting behavioral estrus [27]. Confirming this, cows administered GnRH agonist, dephereline, before AI under heat-stressed conditions exhibited more significant ovulation and conception rates than those administered natural GnRH [28]. Moreover, within 3 h after the OE, the administration of GnRH to dairy cows (250 µg) or a GnRH analog (buserelin, 10 µg) led to a greater amplitude of spontaneous LH peaks, fewer delayed ovulations, and higher P_4_ concentrations after ovulation [29,30]. In dairy cows with high milk production, the use of GnRH markedly improved conception rates. Additionally, injecting cows with a GnRH analog (Gonadorelin, 200 µg) within 5 hours of detecting the OE through the AAM system enhanced conception rates, primarily when inseminations occurred during autumn months, particularly in cows with postpartum health disorders [22]. 

**Table 1:** The application of GnRH analogs at the onset of estrus and AI time for improving ovulation response and conception rates in Holstein dairy cows.

Ref	Animal & Physiological Stage	Groups	Treatment	Fertility Outcomes			Summary and Limitations
				Ovulation%	Conception% /P/AI	Pregnancy Loss%	
[27]	Lactating dairy cows from 3 herds during late summer. Treatment at the observation of estrus.	GnRH (n = 49)	Gonadorelin (Factrel^®^)	-	28.6 (d 45)	0 (after 45d)	GnRH treatment at estrous detection increases P_4_ concentrations, increasing embryo survival and P/AI in treated cows.
		Control (n = 45)	Physiological saline	-	17.7 (d 45)	3.4 (after 45d)	
[29]	Dairy cows (C, n = 86) and heifers (H, n = 23). Treatment at OE, after PGF_2α_- synchronization.	GnRH (n = 54)	100 μg Gonadorelin (Cystorelin@)	-	45.4 (H) 41.9 (C)	-	GnRH administration enhances fertility by affecting P_4_ levels and embryonic survival, possibly linked to a delayed or slow rise in P_4_ concentration after ovulation.
		Control (n = 55)	Physiological saline	-	33.3 (H) 23.2 (C)	-	
[30]	Primiparous and multiparous dairy cows during the summer (S) and winter (W). Treatment within 3 h after estrous detection.	GnRH (n = 157 in S, n = 81 in W)	10 μg Buserelin (Receptal@)	-	51.6 (S) 63.0 (W)	-	The significant effect of GnRH is higher in summer. GnRH can improve overall conception rates in primiparous cows (63.2%) compared to multiparous cows (50.3%) during summer and winter.
		Control (n = 157 in S, n = 75 in W)	Physiological saline	-	35.1 (S) 54.7 (W)	-	
[28]	Heat-stressed lactating dairy cows. Treatment before AI (by 18–20 h) at the end of a 5 d P_4_-based protocol for FTAI.	GnRH (n = 379)	100 μg gonadorelin (Dephereline@)	**Ovulation** **failure** % 11.7	33.5	13.4	Dephereline can serve as an alternative to natural GnRH in inducing ovulation. It reduces the risk of ovulation failure, increasing pregnancy and embryonic survival rates.
		GnRH (n = 369)	Natural GnRH	4.0	24.1	25.8	
[22]	2nd parity cows or more. Treatment within 5 h of the OE confirmed by the AAM system.	GnRH (n = 116)	200 µg Gonadorelin (Gonabreed^®^)	-	56.6	-	GnRH administration within 5 h of the OE, post-AAM determined, increases P/AI in cows during the autumn season.
		Control (n = 117)	Not treated	-	28.5	-	
[31]	Cows were assigned to 6 groups (2 × 2 × 2 factorial experiment) according to injections at 1 h (early) or 12–16 h (late) and AI at 1 h (early) or 12–16 h (late) after detection.	GnRH (n = 208)	100 µg Gonadorelin (Cystorelin@)	In both treatments, AI performed during the early stages of estrus decreased P/AI, ranging from 11% to 16%. Only administration of GnRH early and AI late in estrus returned P/AI to control values (46% vs. 43.3%, respectively).			Inconsistent effects of GnRH on P/AI may be due to differences in GnRH potency or treatment timing. It is not recommended to use GnRH at first services in dairy cattle.
		Control (n = 117)	Physiological saline				
[32]	Lactating cows with average DIM 111.4 d. Treatment at the time of AI.	GnRH (n = 212)	10 μg Buserelin (Receptal@)	-	42.9	13.19	GnRH administration immediately after AI did not improve P/AI ratios and pregnancy survival rates.
		Control (n = 213)	Physiological saline	-	40.8	16.09	
[23]	Primiparous and multiparous cows (>50 d DIM) Treatment at AI after estrous detection by the accelerometer.	GnRH (n = 445)	100 μg Gonadorelin (Fertagyl^®^)	-	26.2 (d 35) 24.9 (d 65)	4.0	GnRH at the AI time after estrous detection by the accelerometer system did not affect fertility in dairy cows. No interaction was detected between treatment and season or AI number.
		Control (n = 534)	Physiological saline	-	22.7 (d 35) 21.8 (d 65)	5.0	
[33]	Lactating cows (110 ± 65 d DIM) during the warm season. Treatment at AI after estrous detection	GnRH (n = 429)	100 μg Gonadorelin (Cystorelin@)	-	30.8	-	GnRH administration at AI diminishes the worm season effect by increasing P_4_ concentrations and conception rates and influencing the incidence of twin pregnancy rates.
		Control (n = 431)	Non treated	-	20.6	-	
[34]	Crossbred dairy cows (Holstein Friesian × Sahiwal) Treatment at AI time.	GnRH (n = 45)	200, 250, or 300 µg Gonadorelin (Ovurelin@)	-	71.11 (overall 3 doses)	-	GnRH at AI time increases conception rates of crossbred dairy cows without the significant effect of different GnRH doses.
		Control (n = 45)	Not treated	-	55.6	-	
[35]	Cows blocked by parity (P1 and P2) detected in estrus using traditional methods of the AAM system. Treatment at the AI time.	GnRH (n = 197)	100 μg Gonadorelin (Gonabreed@)	90.4 ± 3.9	38.6 (P1) 34.5 (P2)	8.2	GnRH had no significant effect on P_4_ on the day of estrous detection and 7 d later. This study did not recommend using GnRH at AI time to improve fertility outcomes in dairy cows.
		Control (n = 201)	Not treated	92.8 ± 3.7	43.3 (P1) 38.4 (P2)	9.8	
[36]	Crossbred cows with estrous signs during winter (W) and summer (S) months. Treatment at AI time after estrous identification	GnRH (n = 33)	10 μg Buserelin (Receptal@)	62.5 (W) 100 (S)	-	-	GnRH administration at AI improves ovulation induction rates, especially during summer.
		Control (n = 87)	Not treated	66.6 (W) 86.2 (S)	-	-	
[37]	Nulliparous dairy heifers. Treatment at AI time after estrous observation.	Sexed semen AI (n = 100)	Not treated	-	49.0	2.04	The fertility output of the conventional semen group is higher than that of the sexed semen groups. However, the fertility of sexed semen can be increased to a comparable level by administering GnRH at AI time.
		Sexed semen AI + GnRH (n = 100)	10 μg Buserelin (Receptal@)	-	54.0	1.85	
		Conventional semen AI (n = 100)	Not treated	-	63.0	3.17	
[38]	Lactating cows (n = 2607) were grouped based on their estrous expression intensity, to high (H) or low (L) estrous expression. Treatment at AI time.	GnRH	100 μg Gonadorelin (Factrel^®^)	**By 7 d:** 98.2 (H) 92.9 (L)	41.3 43.5 (H) 37.8 (L)		GnRH administration at AI time may enhance the fertility of cows with lower estrous expression. However, the improved ovulation rates do not directly cause this relationship. GnRH administration at AI time did not affect the ovulation of cows with lower estrous expression.
		Control	Not treated	**By 7 d:** 92.0 (H) 92.9 (L)	35.7 42.6 (H) 31.0 (L)		
[39]	Seasonal or split calving dairy herds (n = 16) during spring. Treatment at AI time. Cows were blocked by milk protein % and calving-AI interval.	1st AI cows (n = 2344)	250 µg Gonadorelin (Ovurelin@)	Only treated cows at 1st AI with high milk protein (>3.75%) and less than 40 d postpartum increased the herd conception rate by 1.3%			GnRH at AI may enhance oocyte maturation and/or luteal function rather than reducing AI-to-ovulation intervals. GnRH use at AI should be limited to cows most likely to respond.
		2nd AI cows (n = 579)	250 µg Gonadorelin(Ovurelin@)	There were no significant outcomes of GnRH on conception during the 2nd AI.			

AAM: automatic activity monitoring, AI: artificial insemination, d: day, GnRH: gonadotropin-releasing hormone, h: hour, OE: onset of the estrus, n: number, P/AI: pregnant rate to service, P_4_: progesterone, PGF_2α_: prostaglandin F_2α_.

Other studies have administered GnRH at the time of AI and have demonstrated positive impacts on reproductive performance. For instance, utilizing various doses of GnRH at AI has enhanced conception rates in crossbred dairy cows, with no significant difference observed among different GnRH doses [34]. The results from the limited number of published reports on GnRH administration during the period of behavioral estrous onset indicate that the administration of GnRH may have a marked beneficial effect on conception rates under inclement climatic heat conditions. Administering GnRH at AI has been found to mitigate the seasonal variation effect by increasing P_4_ concentrations and improving conception rates [28] or by reducing the risk of ovulation failure, thereby increasing pregnancy rates and embryonic survival in dairy cows [33,36]. Furthermore, in nulliparous dairy heifers, the fertility of sexed semen can be improved to a comparable level by administering GnRH at AI time [37]. In the same context, administering GnRH during AI time in beef cattle has also improved fertility parameters. Administrating GnRH at TAI following 7-day P_4_-based protocols has increased fertility in Bos indicus beef cows [40]. Similarly, supplementing GnRH treatment at TAI within estradiol (E_2_)-P_4_-based protocol has enhanced fertility parameters in Bos indicus cows. However, it did not impact pregnancy loss rates [41].

Conversely, several studies have shown that administering GnRH at OE and AI time does not improve conception rates or fertility in dairy cows [23,31,32,35]. GnRH agonist, gonadorelin, has shown inconsistent effects on P/AI, possibly due to variations in GnRH potency or the timing of administration. Additionally, it is not advisable to administer GnRH during initial services for dairy cattle [31]. Also, administering gonadorelin following estrous detection via the accelerometer system has no significant impact on fertility in dairy cows, regardless of standard or heat-stress conditions [23]. Furthermore, it is not recommended to use GnRH at the time of AI due to its inability to enhance fertility in dairy cows [32,35]. 

## 3. Prevention of Pregnancy Losses by GnRH Treatment

### 3.1. After Artificial Insemination (AI)

About 25% of cattle embryos are non-viable within the first 3 weeks of the gestational period. The maintenance of pregnancy during these initial stages relies on the continuous secretion of P_4_ by a fully functional corpus luteum (CL), and premature luteolysis is considered a primary factor contributing to early embryonic mortality [3]. Over the past 25 years, GnRH-based products have been used as a “holding injection” on the day of AI to enhance the proportion of successful pregnancy, particularly in “repeat breeder” cows [4]. Repeat breeding is commonly characterized as a syndrome with various potential etiologies, including genetic or acquired defects in ova, sperm, or embryos during the initial developmental stages, infections or inflammatory processes, endocrine dysfunctions, nutritional or management deficits, and other factors associated with early embryonic loss or spontaneous abortion [42,43]. Females exhibiting repeated behavioral estrus, with a third or more unsuccessful service, are categorized as “repeat breeders” [2].

The administration of GnRH for repeat-breeding dairy cows has demonstrated beneficial effects, as presented in Table 2. Several studies have indicated that administering GnRH at the time of AI or immediately after AI could positively impact the fertility of repeat-breeder cows [44,45,46,47,48,49]. GnRH administration has been associated with improved pregnancy rates by increasing the concentration of serum P_4_ starting 6 days after administration [45,46,47]. While it has been confirmed that P_4_ levels increase 6 days after GnRH administration in repeat breeders, no positive impact on fertility was observed [48]. Additionally, it has been reported that GnRH administration may increase P_4_ concentrations 2 days earlier in treated cows compared to controls [44].

Furthermore, GnRH administration could increase the diameter of CL and the number of large luteal cells (LLC) within the CL, leading to increased P_4_ concentrations and improved fertility [49]. Previous studies observed consistent findings when administering GnRH or hCG when the CL was developing in the early luteal phase, resulting in enhanced fertility in repeat-breeding cows [50]. Recently, there has been a trend towards maximizing the efficacy of GnRH in treating repeat breeders by administering it along with P_4_, by injection or inserting progesterone-releasing intravaginal devices (PRID) to support early embryonic development while using non-steroidal anti-inflammatory drugs (NSAIDs) to inhibit the synthesis of prostaglandin F2-alpha (PGF_2α_). Studies have found that combined protocols involving GnRH, exogenous P_4_, and tolfenamic acid [51] or meloxicam [52] effectively treat repeat breeders. These protocols support P_4_ concentrations and increase conception rates by up to six times compared to the control group. However, further research in this area is still needed. A meta-analysis of 40 experiments evaluating 27 studies revealed that GnRH administration at the time of AI substantially affected conception rates in repeat-breeding dairy cows (22.5%). In contrast, the positive effect of GnRH was only 5.2% units at the first postpartum AI and 8.0% for the effect of GnRH analogs [53].

Several studies have recommended GnRH administration during the diestrous phase as a potential solution to overcome the impact of repeat-breeding problems in dairy cattle. It has been found that GnRH administration during the diestrous phase (5–7 days post AI) can increase the proportion of cows with an additional CL, which enhances embryo survival [54]. Similar results were observed when GnRH was administered between the 7th and 14th days after AI in dairy cows [55] and lactating buffaloes [56]. Previous research suggested that GnRH administration on day 12 of the previous cycle improves ovulation and conception rates compared to the control group, and it significantly increases P_4_ concentrations in pregnant cows on day 22 [57]. However, the administration of either GnRH or P_4_ (PRID) did not affect the fertility outcomes of repeat breeder cows when used during diestrus (4–18 days post-AI); nevertheless, they increased the P_4_ level in treated cows compared to the control [58]

**Table 2:** Application of GnRH analogs for treating repeat breeder (RB) dairy cows.

Ref	Animal & Physiological Stage	Groups	Treatment	Fertility Outcomes		Summary and Limitations
				Conception % /P/AI	Other Related Parameters	
[2]	Repeat-breeding, lactating cows, 6 herds at their 3rd or 4th service. AI was performed according to the rule (a.m.–p.m.). The 2nd AI for the double AI groups was given 12–16 h after the 1st AI.	Single AI + No treatment (n = 353)	Not treated	32.1	-	GnRH analog administration following single AI caused an increase in the pregnancy rate of repeat breeders compared with nontreated groups. No significant effect was observed when using double AI.
		Single AI + GnRH (n = 406)	100 µg Gonadorelin (Cystorelin@)	41.6	-	
		Double AI + No treatment (n = 364)	Not treated	33.5	-	
		Double AI + GnRH (n = 359)	100 µg Gonadorelin (Cystorelin@)	37.5	-	
[44]	Cows returned to estrus after 2nd AI (95–200 d PP). Treatment at AI time.	GnRH (n = 30)	100 pg Gonadorelin (Cystorelin@)	43.0	**Pregnancy loss %** 33.0	GnRH injection at AI time increases P/AI, and the embryo survives by significantly increasing P_4_ levels 2 d earlier, associated with decreased LH and increased FSH levels at 8 d post-treatment.
		Control (n = 14)	Physiological saline	14.0	50.0	
[45]	Crossbred repeat breeder dairy cows, eligible after 6–8 inseminations but clinically healthy. Treatment at AI time.	GnRH (High dose, n = 55)	20 μg Buserelin (Receptal@)	87.0	**Interval to pregnancy (d)** 84.3 ± 2.2	The high dose of GnRH administration enhances the fertility of repeat-breeder cows by increasing the overall pregnancy rate and decreasing the interval from treatment to pregnancy.
		GnRH (Low dose, n = 40)	10 μg Buserelin	58.0	89.1 ± 3.4	
		Control (n = 42)	Physiological saline	48.0	98.7 ± 4.8	
[46]	Deoni repeat-breeder cows. Estrus was detected by visual observation. AI was performed at an interval of 12 h post-detection.	Control (n = 14)	250 µg Buserelin (Receptal@)	83.3	-	Regardless of the administration day, GnRH administration increases the conception rate in repeat breeder cows.
		2 GnRH at estrus + 12 d after AI (n = 6)		83.3	-	
		2 GnRH at estrus + 14 d after AI (n = 6)		83.3	-	
		Control (n = 6)	Not treated	33.3	-	
[47]	Lactating crossbred cows. Treatments were given at OE. AI after estrous detection.	GnRH (low dose, n = 25)	10 µg Buserelin (Receptal@)	68.0	-	The high dose of GnRH treatment at OE time is more effective by significantly increasing the overall pregnancy rate (after 3 AI) than the control group.
		GnRH (High dose, n = 30)	20 µg Buserelin	80.0	-	
		Control (n = 30)	Physiological saline	46.7	-	
[58]	Repeat-breeder cows between 1st–5th lactation and had 3–6 unsuccessful inseminations. Treatments between 4th–18th d post-AI	G1: GnRH (on d 12, n = 15)	10 µg Buserelin (Receptal@)	20	-	Groups 1 and 3 had significantly higher serum P_4_ concentrations than the other groups. The administration of either GnRH or PRID did not affect the fertility outcomes of repeat-breeder cows.
		G2: P_4_ (d 4 to 11, n = 15)	PRID	26.6	-	
		G3: P_4_ (d 11 to 18, n = 15)		40	-	
		G4: Control (n = 15)	Not treated	20	-	
[57]	Crossbreed repeat-breeders between 2nd–7th lactation failed to conceive in 3 or more inseminations.	G1: GnRH treated-prior to AI (12 d pre-AI, n = 8)	10 µg Buserelin (Receptal@)	62.5	**Ovulation %** 100.0	Administering GnRH on d 12 of the previous cycle or during AI improves ovulation and conception rates (G1 and G2) compared to the control group. It significantly increases P_4_ concentrations in pregnant cows on day 22.
		G2: GnRH treated-at AI (n = 8)	10 µg Buserelin	75.0	100.0	
		G3: GnRH treated-post AI (12 post-AI, n = 8)	10 µg Buserelin	62.5	87.5	
		G4: Control (n = 8)	Physiological saline	25.0	87.5	
[48]	Repeat breeder dairy cows maintained under field conditions. AI at spontaneous estrus. Treatment at AI time.	GnRH (n = 16)	10 µg Buserelin (Receptal@)	43.8	-	GnRH administration for repeat-breeders at AI time did not have an impact on conception rates between groups. GnRH significantly increased P_4_ concentrations on day 6 post-AI.
		Control (n = 17)	Not treated	29.4	-	
[51]	Repeat-breeding cattle aged 3–8 years. Treatment time: GnRH at the time of AIP4 on days 4, 5, and 6 post-AI Tolfenamic acid on day 16, 17, and 18 post-AI	Control (n = 8)	Not treated	12.5	-	The combined protocol (GnRH, exogenous P_4_, and Tolfenamic acid) supports P_4_ concentrations and increases conception rates sixfold over the control group. The combined protocol (GnRH, P_4_, and Tolfeamic acid) is effective in repeat breeder treatment.
		GnRH (n = 8)	20 µg Buserelin	37.5	-	
		GnRH + P_4_ (n = 8)	20 µg Buserelin+ 100 mg Progesterone	50.0	-	
		GnRH + P_4_ + Tolfenamic acid (n = 8)	Buserelin+ P_4_ + 4 mg/kg Tolfenamic	75.0	-	
[52]	Repeat-breeder cows in 5 years suffer from ovulation defects, late P_4_ rise, and premature luteolysis.	G 1: GnRH (4 to 6 h before AI, n = 115)	10 µg Buserelin (Receptal@)	20.0	**Return to estrus %** 51.3	The proportion of cows that returned to estrus after AI did not differ among groups. The pregnancy rate is significantly higher in the G4 (combined protocol) than in other treatments. The combined protocol (GnRH, P_4_, and meloxicam) effectively treats repeat breeders.
		G 2: P_4_ (on d 5 to 7, n = 51)	100 mg P_4_ intravaginally	27.4	52.9	
		G3: Meloxicam (on d 16 to 18, n = 31)	0.5 mg kg^−1^, 24 h^−1^ Meloxicam	22.5	54.8	
		G4: GnRH + P_4_ + meloxicam (n = 98)	All abovementioned treatments	35.7	50.0	
		G5: Control (n = 107)	Not treated	17.7	61.6	
[49]	Repeat-breeder cows at their 3rd service or more. Treatment post-AI.	AI without GnRH (n = 40)	Not treated	42.5	-	Administrating double GnRH with AI increases repeat breeder fertility by increasing CL diameter (23.4 ± 0.4 vs. 16.2 ± 0.4), doubling P_4_ concentration (9.4 ± 0.2 vs. 5.8 ± 0.2), and decreasing E_2_ concentration (1.38 ± 0.18 vs. 2.69 ± 0.25) compared to not-treated group at day 18 post-AI.
		AI + single GnRH (n = 40)	100 µg Gonadorelin (Ovurelin@)	62.5	-	
		AI + double GnRH (n = 40)		70.0	-	
[54]	Repeat-breeder cows (n = 399) and non-RB cows (n = 411). Treatment in the early luteal phase on day 5 to 7 post-AI.	GnRH single dose (n = 270)	100 mg Gonadorelin (Dephereline@)	39.1	**Additional CL %** 57.5	Using a high dose (250 mg) of GnRH in the early luteal phase can overcome the impact of repeat breeding in cows by increasing the proportion of cows with an additional CL, which can enhance embryo survival.
		GnRH 2.5 × dose (n = 271)	250 mg Gonadorelin (Dephereline@)	31.9	52.3	
		Control (n = 269)	Not treated	28.6	18.2	
[55]	Repeat-breeder cows at their 3rd insemination or more (179 ± 38.4 DIM). Treatment is between the 7th and 14th days post-AI.	GnRH (n = 98)	100 μg Gonadorelin (Dephereline@)	64.3	58.2	Administering GnRH agonist between 7th and 14th d after AI improves the potential for a second CL in repeat-breeder pregnant cows, possibly increasing embryo survival
		Control (n = 90)	Not treated	55.5	14.4	
[59]	Holstein dairy cows. Treatment on day 14 post-AI. CIDR insert between (17–24 d).	GnRH + P_4_ (n = 177)	200 µg Gonadorelin (Factrel^®^) + CIDR	37.3	**Return to estrus %** 52.1	Treatment with GnRH +CIDR insert increased pretreatment pregnancy rate but did not affect pregnancy loss.
		Control (n = 170)	Not treated	30.0	59.5	

AI: artificial insemination, CL: corpus luteum, d: day, E_2_: estradiol, FSH: follicle stimulating hormone, GnRH: gonadotropin-releasing hormone, h: hour, LH: luteinizing hormone, n: number, P_4_: progesterone, PR: pregnancy rate, RB: repeat breeder.

### 3.2. In the Early Luteal Phase (Days 4 to 9 after AI)

During the diestrous phase of the estrous cycle, an anovulatory large DF undergoes atresia due to the estrous cycle stage (i.e., luteal phase) during which development occurs. This initiates the luteolytic process through E_2_ produced by the developing follicle. Follicle-induced luteolysis occurs when E_2_ affects the endometrium, whose development and functions are modulated by P_4_, leading to the production and secretion of PGF_2α_ [6,7]. The administration of buserelin (a GnRH agonist) at 3-day intervals between the 12th to 48th days after behavioral estrus prolongs the lifespan of the CL due to sustained luteinization of follicles, resulting in the luteolysis of cells or, in the case of induced ovulation, the development of an accessory CL [60]. A total of 6 to 8 days after the last buserelin injection on day 48, a fully functional DF is developed, leading to luteolysis and subsequent expression of behavioral estrus [13]. Follicular growth is attenuated in the CL-bearing ovary ipsilateral to the pregnant uterine horn. This localized suppression of folliculogenesis and subsequent E_2_ production could enhance the anti-luteolytic functions of the developing embryo by suppressing PGF_2α_ endometrial secretions. Embryonic mortality may occur if the embryo fails to secrete an anti-luteolytic protein, such as the bovine trophoblast protein-1 complex (bTP-1). Improving embryo survival percentages may be achieved by increasing the duration of CL functions, leading to a prolonged period of embryonic development for optimal secretions of bTP-1, inducing an anti-luteolytic effect [61].

Studies summarized in Table 3 demonstrate that administering GnRH analogs during the early luteal phase significantly affects the reproductive performance of dairy cows. Buserelin treatment induced CL-formation between 3 to 6 days after treatment, with large luteal cells, altering CL volume and P4 concentration in cyclic cows compared to acyclic ones. [62]. Gonadorelin administration on day 5 post-AI enhanced accessory CL development and boosted the P_4_ serum concentrations, suggesting improved luteal function [63,64]. Also, GnRH agonist administration via the epidural route on day 7 significantly improved pregnancy rates and overall reproductive performance [65]. Similar results were observed when GnRH and hCG were administered intramuscularly during the 5th–7th day post-AI in dairy cows [66] and beef cows [67]. This administration significantly increased the ovulatory response, induced the formation of CL, and reduced the number of cows returning to estrus compared to control [66]. It has been demonstrated that administering GnRH around the seventh day post-AI aimed at preventing heat stress and excessively high concentrations of P_4_ could potentially enhance the LH release profile in dairy cows. This enhancement in the LH release profile might consequently improve P/AI rates [68].

On the contrary, administering GnRH on day 5 or day 15 post-AI did not result in improved fertility outcomes [69]. In a recent study, administering GnRH and hCG injections 5 days after AI showed a non-significant improvement in pregnancy rates [70]. However, it was noted that GnRH and hCG exhibited beneficial effects by inhibiting the signal pathways of toll-like receptor 4 (TLR-4) and nuclear factor kappa B (NF-κB) while promoting the expression of leukemia inhibitory factor (LIF) and interleukin-1 (IL-1), thereby enhancing uterine receptivity. This inhibition of the immune response in the uterus created a favorable environment for embryo implantation, potentially leading to improved pregnancy rates [70].

These findings collectively highlight the potential of GnRH agonists during the early luteal phase in optimizing reproductive outcomes in dairy cattle, providing valuable insights for enhancing fertility management strategies in the industry.

**Table 3:** Administration GnRH analogs post-AI for improving fertility in dairy cattle.

Ref	Animal & Physiological Stage	Groups	Treatment	Fertility Outcomes		Summary & Limitations
				Conception% /P/AI	Other Related Parameters	
[70]	Holstein cows in their 2nd parity All experimental cows were subjected to the Ovsynch protocol. Treatment on the 5th d post-AI	GnRH (n = 31)	100 μg GnRH	51.6		GnRH and hCG administration 5 d after AI were beneficial for CL function and uterine receptivity. However, the improvement in pregnancy rates was non-significant.
		hCG (n = 58)	1500 IU	53.4		
		Control (n = 52)	Not treated	36.5		
[65]	Multiparous lactating cows with parity ranging from 2 to 4 exhibited estrus spontaneously after their VWP. Treatment at AI time or 7 d post-AI.	Control (n = 38)	Physiological saline	42.1	**Cumulative PR %** 69.7	GnRH administration on day 7 by Ep. route improves the P/AI rate and the reproductive performance of multiparous cows compared to other groups.
		GnRH at AI time (Ep, n = 19)	25 µg of Alarelin (Vetaroline^®^)	36.8	63.2	
		GnRH at AI time (im, n = 12)		16.7	54.6	
		GnRH at d 7 (Ep, n = 13)		61.5	81.8	
		GnRH at d 7 (im, n = 12)		33.3	70.0	
[63]	Holstein cows were synchronized using the Ovsynch protocol. Treatment on the 5th d after AI	GnRH (n = 12)	100µg of Gonadorelin (Cystorelin@)	26.7	-	GnRH injection on day 5 post-AI increases accessory CL development and P_4_ concentrations on d 13 compared to the control group but does not affect the conception rate.
		Control (n = 11)	Physiological saline	24.3	-	
[64]	Lactating dairy cows (n = 158), at 213 ± 112 DIM during summer season. Parameters measured in case of high (>39.7 °C) and low (<39.7 °C) rectal temperature. Treatment on the 5th d after AI.	GnRH (n = 55)	100 µg Gonadorelin (Cystorelin@)	36.8 (<39.7 °C) 17.8 (>39.7 °C)	-	GnRH or hCG administrations induce accessory CL and increase P_4_ concentration, only improved pregnancy in cows with low rectal temperature (<39.7 °C), indicating that high temperature at AI is more harmful than beneficial.
		hCG (n = 51)	2500 IU	32.8 (<39.7 °C) 24.4 (>39.7 °C)	-	
		Control (n = 52)	Physiological saline	10.1 (<39.7 °C) 15.2 (>39.7 °C)	-	
[66]	AAM system detected Lactating Holstein cows (H) and lactating Jersey cows (J) in estrus from 27 to 50 DIM. Treatments between days 5 and 7 of the estrous cycle.	GnRH (Holstein, n = 116; Jersey, n = 75)	86 mg Gonadorelin (Fertagyl^®^)	35.4 (H) 39.6 (J)	**Return to estrus** 75.0	The administration of GnRH and hCG significantly increased the ovulatory response, induced the formation of CL, and decreased cows returning to estrus compared to control cows.
		hCG (Holstein, n = 127; Jersey, n = 69)	3300 IU hCG	31.5 (H) 54.3 (J)	74.0	
		Control (Holstein, n = 111; Jersey, n = 66),	Physiological saline	34.3 (H) 38.6 (J)	86.0	
[68]	Lactating dairy cows at 108.2 ± 2.3 DIM during worm (W) and cool (C) season Treatment on day 7 post-AI	GnRH (gonadorelin, n = 56)	100 µg Ovarelin^®^	33.7 (W) 49.0 (C)	**Pregnancy loss** 2.1 (W) 4.3 (C)	Regardless of the GnRH type used, GnRH aimed at preventing heat stress and excessively high levels of P_4_ around the time of GnRH treatment could potentially enhance the LH release profile in dairy cows and consequently improve P/AI rates.
		GnRH (buserelin, n = 52)	10 µg Receptal^®^	31.9 (W) 38.2 (C)	3.7 (W) 6.6 (C)	
[69]	Multiparous cows were treated with one luteolytic dosage of PGF_2α._ Treatment on days 5 or 15 after TAI.	GnRH on d 5 (n = 214)	100µg of Gonadorelin (Cystorelin@)	47.7	19.6	GnRH administration on days 5 or 15 post-AI did not improve the fertility outcomes. Administration of GnRH on days 5 and 15 had a lower fertility outcome.
		GnRH on d 15 (n = 209)		43.5	13.2	
		GnRH on d 5 + d 15 (n = 212)		36.8	23.0	
		Control (n = 196)	Physiological saline	44.4	19.5	
[71]	Lactating Holstein cows Treatment on days 11 to 14 after 1st AI.	GnRH (n = 34)	8 µg Buserelin (Receptal^®^)	34.3	-	The administration of GnRH did not increase pregnancy rates.
		Control (n = 35)	Physiological saline	37.3	-	
[72]	Holstein cows. Treatment on day 12 post-AI.	GnRH (Full dose, n = 238)	21 µg Buserelin (Receptal^®^)	65.3	-	The doses of 10.5 µg or 21.0 µg of GnRH were equally effective when administered on day 12 post-AI
		GnRH (Half dose, n = 202)	10.5 µg Buserelin	67.7	-	
		Control (n = 91)	Not treated	49.5	-	
[73]	Holstein–Friesian dairy cows. (n = 103) at day 42 of VWP. Treatment on day 12 post-AI.	GnRH (n = 49)	50 µg Gonadorelin (Gonavet^®^)	59.6	-	Using a GnRH agonist on day 12 post-AI did not enhance the reproductive performance of dairy cows.
		Control (n = 54)	Not treated	59.1	-	
[74]	Multiparous Holstein dairy cows. Treatment on day 14 post-AI.	GnRH (n = 25)	0.02 mg Buserelin (Receptal^®^)	**Pregnancy relative rate** = 1.73	**Days open** 76–153 d	GnRH analog administration significantly increased reproductive parameters and total milk yield for the GnRH-treated cows compared to control.
		Control (n = 20)	Not treated		90–180 d	
[75]	Holstein cows in two dairy farms Treatment on day 21 after a pre-enrollment AI.	GnRH (n = 290)	100µg of Gonadorelin (Cystorelin@)	70.9 (d 21) 27.0 (d 42)	**Pregnancy loss %** 55.5 (d 21–28) 9.8 (d 28–42)	The administration of GnRH on day 21 following AI before pregnancy detection does not alter the reproductive performance of dairy cows between days 21 and 42.
		Control (n = 295)	Physiological saline	73.0 (d 21) 26.8 (d 42)	50.6 (d 21–28) 24.1 (d 28–42)	
[11]	Holstein cows in their 3rd lactation carry live singletons (n = 1054) or unilateral twins (n = 379) Treatment on days 28 to 34 at the time of pregnancy diagnosis.	GnRH (low dose, n = 480)	100 μg Gonadorelin (Cystoreline^®^)	52.7	12.4	A high dose of GnRH administered at the time of pregnancy diagnosis did not affect the pregnancy rate. In contrast, GnRH treatment reduced the proportions of pregnancy loss.
		GnRH (high dose, n = 482)	200 μg Gonadorelin	56.3	12.3	
		Control (n = 471)	Physiological saline	56.1	17.2	

AAM: automated activity monitoring, AI: artificial insemination, CL: corpus luteum, d: day, DIM: days in milk, GnRH: gonadotropin-releasing hormone, hCG: human chorionic gonadotropin, LH: luteinizing hormone, n: number, P/AI: pregnant rate to service, P_4_: progesterone, VWP: voluntary waiting period.

### 3.3. During the Luteal Development (Days 11 to 14 after AI)

Several studies have investigated the impact of administering a GnRH agonist post-insemination during the luteal phase of the estrous cycle on conception percentages (Table 3). In dairy cows, the administration of 10 µg buserelin between days 11, 12, and 13 post-AI resulted in an enhanced pregnancy rate (72.4%) compared to non-treated cows (60.9%) [76]. Additionally, treatment with 10 µg buserelin decreased the percentage of return to service intervals by 22 days compared to non-treated cows, suggesting that the GnRH agonist delayed luteolysis by influencing the dynamics of follicular functions [76]. This delay was beneficial for cows at risk of embryonic losses due to luteolysis during the initial stages of gestation [77]. Interestingly, even without a viable embryo in the uterine lumen, follicular development was not required for pregnancy maintenance, as demonstrated by successful pregnancies achieved through priming with E_2_ followed by progestin replacement after ET into ovariectomized cows [8]. In this context, doses of 10.5 µg or 21.0 µg of GnRH were equally effective when administered on day 12 post-AI, leading to increased reproductive performance in dairy cows [72]. Additionally, administration of GnRH analog on day 14 significantly improved reproductive parameters and total milk yield compared to the control group [74].

Conversely, some studies reported no beneficial response to administering GnRH agonists during the post-AI period. For instance, there were no significant effects on conception rates in experiments involving synchronization using a GnRH-PGF2α based program with additional administration of buserelin (8 µg) 12 days after insemination [9,10]. Similarly, using a GnRH agonist on days 12 to 14 after AI did not enhance the reproductive performance of dairy cows [71,73].

It was hypothesized that estrous synchronization treatment regimens using PGF_2α_ might lead to luteal phases of shorter duration, potentially reducing fertility [78]. Similar results were observed in lactating cows during the summer when GnRH agonist was administered post-AI, showing no significant impact on conception rates [10]. In this case, the lack of effect may be attributed to heat stress causing early embryonic mortality, potentially counteracting any beneficial effects of the GnRH agonist treatment on embryo survival. Therefore, when evaluating fertility responses, it is crucial to consider environmental conditions and reproductive management practices applied when implementing hormonal treatment regimens for FTAI.

### 3.4. During Early Pregnancy Diagnosis (around Day 30 after AI)

In contrast to previous findings [79], GnRH administration around 30 days post-AI (early pregnancy diagnosis period) does not impact embryonic loss during the gestational period, particularly in animals with a single fetus (Table 3). However, in cases of unilateral twin pregnancies, GnRH administration results in more remarkable survival of the two fetuses and an increased percentage of pregnancy in cows with two-fetus pregnancies. These results align with another study [11] where GnRH was administered between days 28 and 34 after insemination. Additionally, administering GnRH injection between days 26 and 71 after insemination did not affect the percentage of pregnancy loss [80]. The same results were found after administration of GnRH on day 21 following AI, before pregnancy detection, and it was also demonstrated that GnRH does not alter the reproductive performance in dairy cows between 21 and 42 days post-AI [75].

Similarly, the administration of GnRH via a deslorelin implant on day 27 after insemination did not affect the percentage of cows pregnant. However, there was a lower incidence of pregnancy loss between days 45 and 90 after insemination when accessory CL was induced with this treatment [81]. Doubling the GnRH dose at the time of pregnancy diagnosis did not affect pregnancy percentage compared to the administration of a single dose of GnRH [11].

## 4. GnRH and Cystic Ovarian Disease (COD) “Follicular Cyst”

A cystic follicle is a prevalent reproductive dysfunction in dairy herds, particularly in cows producing large quantities of milk during the early postpartum period [82]. This condition occurs when a large ovarian follicle persists in the functional state in the ovary for an extended period without a CL. It is defined as an anovulatory follicle in the ovary for 7 to 10 days with a diameter ranging from 1.7 to 2.5 cm or larger [83]. Terms such as ovarian cyst (OC), cystic ovarian degeneration, cystic ovaries, nymphomania, and virilism are used interchangeably to refer to this dysfunction [38]. The interaction between genetic, phenotypic factors, and environmental stressors may lead to a lack of ovulation by affecting the hypothalamic-pituitary axis and inhibiting the secretion of GnRH and/or gonadotropins, resulting in follicular cyst syndrome [4]. The presence of follicular cysts may lead to economic losses, including prolonged calving intervals, lower pregnancy rates, decreased net calf crop percentages, and higher herd culling percentages [84].

Due to its small molecular weight, GnRH administration is the preferred treatment for cows with a cystic ovarian follicle. This results in a lack of immune reactions compared to multiple treatments with hCG and LH from other species [85]. GnRH and its analogs have been effectively utilized to treat cystic follicular syndrome [12]. The first report of GnRH treatment for cows with a follicular cyst indicated 70% to 95% effectiveness 18 to 25 days after treatment [86]. GnRH administration is routinely used to induce follicular dynamic alterations in cows with cystic follicles [13]. In response to GnRH treatment, LH secretion is stimulated, reaching a maximum concentration within 90 to 150 min after treatment. This leads to luteinizing the follicular cysts and the consequent expression of behavioral estrus within 4 weeks post-treatment. Although ovulation from the cystic follicle may not occur in response to GnRH, there may be ovulation from other follicles at treatment time [82].

Several studies recommend using GnRH or GnRH agonists for the treatment of ovarian cysts (Table 4). It has been found that a single intramuscular injection of buserelin at a dose of 10 or higher is as effective as 10,000 IU hCG, making it a recommended treatment for ovarian follicular cysts in cows [87]. Additionally, another study found that the administration of GnRH decreased the prevalence of cystic ovarian follicles by day 7 and day 21 compared to the control group [88]. There has also been a recommendation to treat cows with 21 µg of buserelin intramuscularly for beneficial therapeutic effects in dairy cows [89]. The administration of lecirelin via epidural administration has shown a beneficial impact on reproductive parameters in treating follicular cysts [90]. The intramuscular administration of lecirelin (0.1 mg) has been reported as an effective therapy for ovarian cysts in dairy cows that are over 90 days postpartum following confirmation of the presence of a cystic follicle (>20 mm in diameter) [91]. In a comparative study [92], a single administration of buserelin at a concentration of 20 µg had an equal therapeutic effect in cows with ovarian cysts compared to a single injection of 200 µg of fertirelin, attributed to the higher potency of buserelin in inducing the release of LH and follicle-stimulating hormone (FSH) from the pituitary and luteinizing the follicular cyst. Conversely, there were no detectable effects on the values for fertility variables as a result of administering two doses of buserelin and gonadorelin (GnRH agonists) to cystic cows in postpartum periods [93].

Combining GnRH agonists with other hormones, such as PGF_2α_ and P_4_ (P_4_ intravaginal device, CIDR) for altering the follicular dynamic results in a shorter period of OC effects on reproductive functions [13]. The recommendation for administering PGF_2α_ at 7 to 10-day intervals after GnRH administration has shown beneficial therapeutic effects in cyst remission, significantly improving values for reproductive variables, including intervals of return to estrus, increased ovulation, and pregnancy percentages [43]. Implementing the ovsynch treatment regimen has also been a beneficial therapy protocol for COD in dairy herds [94]. Modifying breeding programs, such as imposing the ovsynch treatment regimen for OC syndrome, may lead to greater reproductive performance [95].

**Table 4:** Therapeutic effect of GnRH injection on cystic ovarian disease (COD) in dairy cattle.

Ref	Animal & Physiological Stage	Treatment	GnRH Analog	Fertility Outcomes			Summary and Limitations
				Conception% /P/AI	Other Related Parameters		
[96]	Holstein cows have both types of cysts (follicular or luteal) between days 45 and 60 postpartum.	GnRH for follicular cyst (n = 80)	Intravaginal, 20 µg Buserelin (Receptal^®^)	40.0	**Recovery time** 24.5 ± 4.0 d		Regarding the conception rate, no significant differences were found between the two types of cysts. Luteal cysts have a significantly shorter recovery time than follicular cysts, indicating faster recovery for the luteal type.
		GnRH for luteal cyst (n = 40)		40.7	16.1 ± 2.1 d		
[87]	Holstein cows with follicular cysts and low milk P_4_ concentrations 1 week before treatment.	GnRH (n = 18)	10 µg Buserelin (Receptal^®^)	44.4	**Disappearance %** 61.1		GnRH injection is as effective as hCG. GnRH is recommended for the treatment of COD in dairy cows.
		hCG (n = 17)	10,000 IU hCG (Gestron^®^)	47.0	82.3		
[88]	Holstein cows with a DF (12–25 mm, without CL > 10 mm) Treatment during the 5th week postpartum.	GnRH (n = 118)	10 µg Buserelin (Receptal^®^)	42.3	**COFs incidence %** 7.6 (d 7) 11.0 (d 21)		The administration of GnRH led to a decrease in the prevalence of COFs by days 7 and 21 compared to the control. There was no improvement in fertility outcomes, including (calving-to-conception interval, first-service conception rate, and NSC).
		Control (n = 119)	Physiological saline	41.3	16.8 (d 7) 21.8 (d 21)		
[89]	Holstein cows were diagnosed with both types of cysts (follicular or luteal)	GnRH for follicular cyst (n = 80)	21 µg Buserelin (Receptal^®^)	67.7	**Estrous rate** 77.5		GnRH administration significantly increased the estrous rate and conception rate of cows treated for follicular cysts compared to PGF_2α_-treated cows for follicular cysts. There is no significant difference between groups in estrous and conception rates for cows with luteal cysts. GnRH is recommended for treating follicular cysts, while PGF_2α_ is recommended for treating luteal cysts in dairy cows.
		GnRH for luteal cyst (n = 69)		47.5	72.4		
		PGF_2α_ for follicular cyst (n = 78)	25 mg PGF_2α_ (Dinoprost^®^)	60	55.1		
		PGF_2α_ for luteal cyst (n = 101)		56.9	77.2		
[97]	Cystic Friesian lactating cows received several treatments:	GnRH (n = 45)	Intravaginal 20 µg Buserelin (Receptal^®^)	45.2	**Cure %** 64.0	**Time** 17.9 d	Based on the findings, GnRH could be considered the first treatment of COD cows, considering efficacy, costs, and easy administration.
		hCG (n = 33)	hCG (3000 IU i.m.)	47.8	66.0	17.7 d	
		P_4_ (n = 19)	PRID (for 10 d)	46.2	63.0	19.7 d	
[91]	Cystic lactating cows above 90 d postpartum, between 2nd and 5th lactations.	GnRH (n = 12)	0.1 mg Lecirelin (Dalmarelin^®^)	-	**CL formation** 75.0		GnRH agonist was adequate for treating ovarian cysts in dairy cattle. However, 25% of the cows in the treated group remained with the cyst.
		Control (n = 8)	Physiological saline	-	12.5		
[98]	Lactating cows with ovarian cysts. Treatment between days 40 and 80 after parturition.	G1: GnRH and PGF_2α_ (n = 54)	20 µg Buserelin (Receptal^®^) + 25 mg Dinoprost^®^	62.96	**Disappearance %** 92.6		In both treated groups, the rate of cysts disappearance was significantly higher than the control group. The pregnancy tendency rate was higher in both treated groups than the control cows.
		G2: GnRH alone (n = 42)	20 µg Buserelin (Receptal^®^)	61.90	95.24		
		G3: Control (n = 22)	Not treated	54.55	72.73		
[93]	Dairy cows with COD aging between 3 to 5 years Treatment on the 14th and 21st after parturition.	G1: GnRH (Buserelin, n = 31)	200 µg Buserelin (Receptal^®^)	43.9			There were no significant differences in fertility parameters when using two doses of different GnRH analogs at 14 and 21 d after parturition.
		G2: GnRH (Cystorlin, n = 14)	100 µg Gonadorelin (Cystorlin^®^)	45.6			
		G3: Control (n = 30)	Not treated	45.5			
[99]	Holstein female (cows and heifers) Cows were treated on day 6 or 7 of the subsequent cycle when a DF was present.	GnRH (Gonadorelin, n = 11)	100 mg of Gonadorelin (Cystorlin^®^)		**Disappearance %** 73.0		Based on these results, even a half dose of Lecirelin can be recommended in an ovulation synchronization program for cattle. This can help to synchronize the emergence of a new cohort of follicles.
		GnRH (Lecirelin 25, n = 11)	25 mg of Lecirelin (Dalmarelin^®^)		82.0		
		GnRH (Lecirelin 50, n = 12)	50 mg of Lecirelin (Dalmarelin^®^)		100.0		
		GnRH (Buserelin, n = 12)	10 mg of Buserelin (Receptal^®^)		100.0		

AI: artificial insemination, CL: corpus luteum, COD: cystic ovarian disease, COFs: cystic ovarian follicles, d: day, DF: dominant follicle, GnRH: gonadotropin-releasing hormone, hCG: human chorionic gonadotropin, i.m.: intramuscularly, IU: international unit, n: number, NSC: number of services per conception, P_4_: progesterone, PGF_2α_: prostaglandin F2α, PRID: progesterone releasing intravaginal device.

## 5. Postpartum Ovulation Induction

The interval from parturition to the resumption of ovarian activity is essential in the reproductive cycle of dairy cows, marked by distinct physiological changes and significant commercial implications. Several factors contribute to the occurrence and persistence of anovulatory periods during the postpartum phase, including diminished LH release in late gestation, nutritional status during the peripartum period, mainly if there is a negative energy balance, and the influence of suckling stimulus in lactating cows nursing calves [14].

Ensuring the complete involution and infection-free status of the uterus and the timely resumption of estrous cycles (within 50 to 60 days postpartum) are critical aspects of managing fertility and optimizing reproductive performance in dairy herds [74]. Monitoring reproductive processes is particularly vital in seasonal calving systems, where the window for calving and achieving pregnancy is time limited. The aim is to ensure that each cow produces a calf annually [100].

Hormonal treatment plays a significant role in synchronizing reproductive functions and inducing ovulation during the early postpartum period. The presence of follicular wave patterns and follicles larger than 9 mm in diameter in the ovaries prompts the administration of GnRH. This induces an LH surge, leading to ovulation from the DF during the early postpartum period in dairy cattle [14]. The subsequent reduction in E_2_ concentrations, ovulation triggered by GnRH treatment, and the rise in P_4_ concentrations from CL development contribute to improved reproductive outcomes [62]. Administering GnRH during the early postpartum period (between days 10 and 14) proves beneficial in enhancing the reproductive performance of cows that have experienced an abnormal puerperium [101]. While administering this treatment during the early postpartum period (within days 11 to 25) successfully reduces the postpartum to the estrous interval in cows, it does not significantly impact their subsequent reproductive efficiency [102]. Multiple studies involving dairy and beef cattle have consistently shown that using GnRH alone or followed by PGF_2α_ administration can enhance behavioral estrous responses and pregnancy rates. Significantly, these studies indicate a reduction in embryonic/fetal losses when these treatment regimens are applied [103,104,105].

GnRH administration has improved reproductive efficiency by reducing the calving-to-conception period and the number of inseminations per conception [106]. GnRH treatment during the postpartum period improves ovarian functions early in the postpartum period, leading to a shorter calving-to-estrous interval, approximately 44.6 ± 9.2 days in GnRH-treated cows compared to untreated ones (110.4 ± 50.4) [107]. Single administrations of GnRH analogs between days 21 and 31 postpartum induce LH surges and ovulation, showcasing their potential to improve reproductive outcomes [108]. Administering GnRH or hCG to anovulatory anestrous cows nine days before mating increases ovulation rates and may lead to higher pregnancy rates after the first insemination than untreated cows. However, it may not significantly impact overall reproductive performance [109] and the use of GnRH or hCG treatment did not show significant effects on overall reproductive performance. Therefore, the authors proposed the possibility of a positive impact on reproductive performance if these treatments are administered earlier [109]. Additionally, administering two doses of GnRH at a 10-day interval may induce ovulation and lead to an estrous cycle [110].

Sub-optimal fertility may result from inseminating at the first estrus postpartum due to the formation of abnormal CL. Nevertheless, a short-term treatment with a P_4_ implant may stimulate follicular maturation. Similarly, GnRH treatments may positively influence follicular maturation by increasing P_4_ concentrations in anestrous cows. The induction of typical estrous cycles with favorable fertility outcomes in anestrous cows can be achieved by injecting PGF_2α_ 6 days after GnRH treatment, reducing P_4_ concentration within 24 h [68]. Therefore, in both estrous cyclic and acyclic cows, implementing GnRH-PGF_2α_ treatment regimens can synchronize ovarian functions, leading to average fertility outcomes [111]. Moreover, ovulation induction in acyclic cows treated with GnRH 7 days before administering PGF_2α_ (known as Select-Synch) showed 38% and 49% ovulation rates in two experiments, respectively [112]. In fixed-time embryo transfer (FTET), the transfer of embryos from donor females to recipient females is scheduled at a predetermined time, usually synchronized with the estrous cycle of the recipients.

## 6. In Vivo and In Vitro-Derived Embryos for Transfer into Recipient Cows

Embryo transfer (ET) is an essential biotechnology in animal production employed to breed animals with superior genetics. Despite the increasing application of ET in cattle, conclusive results regarding factors affecting its efficacy remain elusive [113]. One significant factor that can impact outcomes is the accurate detection of estrus. To overcome this limitation, breeding protocols designed originally for FTAI, which synchronize the time of ovulation among females, can be adapted for FTET applications in recipient cows [114].

Additionally, the functionality of the CL plays a vital role in maintaining pregnancy post-ET. The concentration of serum P_4_ is correlated with CL functions, and P_4_ significantly affects conception percentages and in-utero embryo development [113,115]. P_4_ modifies gene expression for uterine-derived proteins, facilitating embryo development and bTP-1 production. A higher P_4_ concentration post-AI is essential for sustaining pregnancy and promoting optimal conceptus growth [116,117,118]. To address luteal insufficiency during ET, several hormonal therapies, such as exogenous P_4_, hCG, or GnRH/GnRH analogs, have been identified as potential solutions.

Previous studies reported varied outcomes regarding the effects of exogenous P_4_ on pregnancy percentages following ET. While some studies suggest a modest positive effect [119,120], others indicate higher embryonic loss and lower pregnancy percentages with P_4_ supplementation four days before ET [121].

Furthermore, numerous studies confirm that administering hCG during ET processes leads to desirable outcomes. Treatment with hCG at the time of ET results in the development of accessory CL, increased P_4_ concentrations, and higher pregnancy rates [16,17]. Additionally, early-stage embryonic losses are reduced after ET. Treatment with hCG following a P_4_-based treatment regimen for estrous synchronization increases pregnancy percentages significantly in lactating cows receiving in vitro-derived embryos by up to 55% [18]. Similar benefits are observed in beef cattle, where hCG treatment during ET regimens results in the development of CL and a higher Doppler score associated with an increased pregnancy percentage [19].

Moreover, the administration of GnRH analogs has been suggested to improve outcomes in ET. GnRH during the diestrous phase induces the development of an accessory CL by either ovulation or luteinization of the DF in recipient cows’ ovaries [122]. A single injection of GnRH on day 5 of the estrous cycle induces ovulation and accessory CL development, resulting in higher P_4_ concentrations and increased pregnancy percentages in heifers receiving in vitro-derived blastocysts [123]. Administering GnRH analogs during ET procedures in cattle increases pregnancy percentages at both days 30 and 60 (45.8% and 43% compared with 40% and 37% in the control group, respectively), with a reduction in pregnancy loss (4% compared with 7% in the control group) [124]. Similarly, the pregnancy rate per FTET is markedly enhanced by up to 55% at day 35 of gestation by treating recipient females with GnRH analogs on the day of ET [125]. Beneficial effects of GnRH on pregnancy percentage were observed when administered on day 11 of gestation with ET [126], while contrasting findings emerged for administration on days 11 and 14 [127,128]. A recommendation has been made for combining a single-dose GnRH injection and a P_4_ implant to mitigate embryonic losses in cows receiving cryopreserved embryos into recipient females [119].

## 7. GnRH Nano-Drug Delivery System

Using nano-drug delivery systems presents a promising approach designed to extend the half-life of bioactive components, facilitate their transport through biological barriers, and ensure sustained delivery to target organs. This innovative method can potentially enhance cellular uptake of bioactive components, allowing for smaller doses than conventional administration, benefiting substances such as hormones, peptides, and drugs [129,130].

While studies have explored the impact of nano-GnRH agonists on reproductive performance and fertility in various farm animals, research on their effects in dairy cattle is currently limited. In rabbits, Hassanein et al. [20] utilized a GnRH agonist (buserelin) loaded on chitosan nanoparticles to induce ovulation, successfully reducing the conventional dose by half without compromising fertility. Similarly, in rabbits, applying chitosan-dextran sulfate GnRH (buserelin) nanoparticles intravaginally with seminal doses for ovulation induction reduced the conventional GnRH agonist dose without compromising reproductive performance variables [21]. During the low-breeding season in buffalo cows, where reproduction is typically reduced, the administration of nano-GnRH analog (ovurelin) as a modified ovsynch treatment regimen led to a 50% reduction in the conventional dose, with beneficial effects occurring on fertility and ovarian activity in anestrous cows [131]. Applying the same methodology in goats, developing a nano-GnRH analog (gonadorelin) allowed for a reduction of hormonal dose by up to 75%, maintaining fertility and prolificacy without adverse effects [132]. Additionally, administering nano-GnRH agonist (ovurelin) as part of the ovsynch treatment regimen proved efficacious in enhancing ovarian activity, blood flow, steroid synthesis (E_2_ and P_4_), and CL function during estrous synchronization in goats [133].

Despite some positive benefits of nano-GnRH treatments on reproductive performance in farm animals, further research is necessary to comprehensively understand their effectiveness, safety, and optimal dosing regimens. Additionally, economic cost and feasibility considerations for producing and administering nanoparticles must be considered.

## 8. Conclusions

In conclusion, GnRH and its agonists have important and diverse functions in optimizing bovine reproductive practices. GnRH exhibits diverse applications, from preventing early embryonic mortality to managing cystic ovarian disease and enhancing reproductive efficiency. This review article discusses the functions of hypothalamic GnRH in reproductive processes and various applications of GnRH, such as preventing embryonic mortality, regulating ovarian follicular wave dynamics, inducing ovulation, managing cystic ovarian disease, and utilizing GnRH as a nano-formulation for enhancing reproductive efficiency. Further research and development in this field will contribute to advancing reproductive efficiency and health management in farm animals.

## Data Availability

Data sharing is not applicable.
